# Proprioceptive precision is impaired in Ehlers–Danlos syndrome

**DOI:** 10.1186/s40064-015-1089-1

**Published:** 2015-07-07

**Authors:** Holly A Clayton, Stephanie A H Jones, Denise Y P Henriques

**Affiliations:** Centre for Vision Research, York University, Toronto, Canada; Department of Psychology, York University, Toronto, Canada; School of Health and Human Performance, Dalhousie University, Halifax, Canada; School of Kinesiology and Health Science, York University, 4700 Keele Street, Toronto, ON M3J 1P3 Canada

**Keywords:** Proprioception, Ehlers–Danlos syndrome, Joint hypermobility, Connective tissue disorders, Proprioceptive sensitivity, Chronic pain, Joint position sense

## Abstract

It has been suggested that people with Ehlers–Danlos syndrome (EDS), or other similar connective tissue disorders, may have proprioceptive impairments, the reason for which is still unknown. We recently found that EDS patients were less precise than healthy controls when estimating their felt hand’s position relative to visible peripheral reference locations, and that this deficit was positively correlated with the severity of joint hypermobility. We further explore proprioceptive abilities in EDS by having patients localize their non-dominant left hand at a greater number of workspace locations than in our previous study. Additionally, we explore the relationship between chronic pain and proprioceptive sensitivity. We found that, although patients were just as accurate as controls, they were not as precise. Patients showed twice as much scatter than controls at all locations, but the degree of scatter did not positively correlate with chronic pain scores. This further supports the idea that a proprioceptive impairment pertaining to precision is present in EDS, but may not relate to the magnitude of chronic pain.

## Background

Ehlers–Danlos syndrome (EDS) is a group of genetic connective tissue disorders that can afflict up to 2% of individuals (Castori 2012). EDS is currently classified into six major types (classic, hypermobility, vascular, kyphoscoliosis, arthrochalasia and dermatosparaxis), with the classic and hypermobility types being the most common (Beighton et al. 1998). Most forms of EDS affect collagen throughout the body; some directly impact its structure (such as with classic type), while others alter proteins that interact with collagen. However, the genetic etiology for the most common type of EDS—hypermobility type—is still unknown (Castori 2012). Although symptomatology can vary across, or sometimes within, each of the subtypes, the most common variants (classic and hypermobility) often present with hypermobile joints, atypical skin (possibly doughy, stretchy, saggy, atrophic, thin, translucent, and/or fragile), chronic pain, chronic fatigue, dysautonomia, developmental delays, poor wound healing, and may bruise easily (Sacheti et al. 1997; Beighton et al. 1998; Gazit et al. 2003; De Paepe and Malfait 2004; Malfait et al. 2010; Rombaut et al. 2010b; Castori 2012). Clinicians also report that these patients are generally clumsy in nature, substantiating the suggestion that EDS patients may have proprioceptive impairments (Rombaut et al. 2010a). However, little is known about the exact nature of these sensory impairments; there are only a few studies that have attempted to explore proprioceptive abilities in EDS patients, or those with hypermobility syndrome (HMS) (Hall et al. 1995; Sahin et al. 2008; Rombaut et al. 2010a). Because the genetic etiology of HMS is still unknown, in addition to it having a virtually indistinguishable presentation from EDS hypermobility type (Tinkle et al. 2009), we will consider results obtained from HMS studies to be applicable to EDS hypermobility type.

Hall et al. (1995) was among the first to study proprioception in HMS patients by examining the knee joint. Using a threshold-detection paradigm in which participants indicated when they could feel movement in the knee joint and were asked to report the direction of the movement, the study revealed that hypermobile subjects showed significantly higher threshold detection levels at knee flexion angles of 5° and 30° in comparison to age and gender-matched control subjects. These findings are similar to those reported by Sahin et al. (2008) who found that HMS patients had significantly higher absolute angular errors than age and gender-matched control subjects during a knee joint matching task. Rombaut et al. (2010a) later compared proprioceptive abilities and vibratory perception sense in EDS hypermobility type patients to those of age and gender-matched controls. Exploring proprioception in the knee and shoulder using both an active and passive joint matching paradigm, they found that EDS patients showed significantly larger angular errors in joint matching at the knee joint, but not at the shoulder. However, they also found that EDS patients could detect tactile stimuli just as well as controls (using vibratory perception threshold at these same joints), suggesting that cutaneous receptors in the skin may not be contributing to the observed proprioceptive deficit in EDS; it could be that tendon and joint receptors are the most likely contributors to the proprioceptive impairment found in EDS (Hall et al. 1995; Sahin et al. 2008; Rombaut et al. 2010a).

Recently, a study in our lab explored proprioceptive localization of the hand in the two most common variants of EDS, classic and hypermobility types (Clayton et al. 2013). We found that, although EDS patients were just as accurate as controls in estimating the location of their unseen hand, they were less precise when tested in the peripheral workspace compared to healthy controls. Specifically, they showed a greater just-noticeable difference compared to healthy controls, in that their unseen hand had to be further left or further right before they were as certain of its position relative to a visual reference as the controls. Moreover, we found a significant correlation between the magnitude of joint hypermobility (Beighton scores) and the magnitude of this proprioceptive deficit, such that those who were the least precise were those who had the highest Beighton scores. We were not able to detect any significant differences between the two types of EDS.

Given that EDS patients, who exhibit joint hypermobility, seem to have some proprioceptive impairments that vary across the workspace (Clayton et al. 2013), our goal was to further examine proprioceptive sensitivity in EDS across a greater number of workspace locations, and to see how they differ compared to healthy controls. Again, we wanted to examine proprioceptive localization of the hand, a body part in which movements need to be monitored with precision in order to interact with the environment. While joint matching tasks, or motion threshold detection paradigms, are commonly used to examine proprioception in patients exhibiting joint hypermobility (Hall et al. 1995; Sahin et al. 2008; Rombaut et al. 2010a; Smith et al. 2013), we used a paradigm which allowed us to precisely place the hand at a greater number of workplace locations in order to obtain very sensitive measures of hand proprioception (Jones et al. 2012b). Here, we assess proprioceptive localization of the non-dominant left hand by having subjects reach to its unseen location with their visible right hand. Since many EDS patients suffer from chronic pain (Sacheti et al. 1997; Voermans et al. 2010), and proprioceptive deficits have been observed in other chronic pain populations (Gill and Callaghan 1998; Knox et al. 2006; Lewis et al. 2010; Tsay et al. 2015), we considered the possibility that pain might be influencing our results, as well. Therefore, we quantified each patient’s chronic pain to explore whether those with the most pain also have the worst proprioception.

## Results

### Accuracy

**Figure 1:**
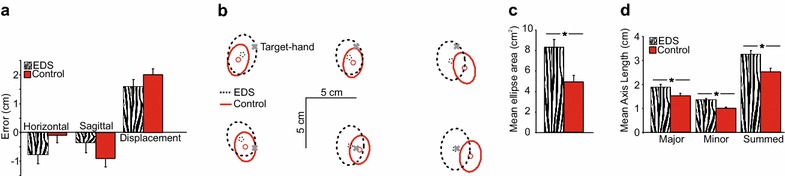
**a** Average horizontal and sagittal reach endpoint errors. *Error bars* reflect standard error of the mean. **b** Average two-dimensional errors (centre of ellipses, represented by *circles*) and precision of reach endpoints (95% error ellipses) at each target-hand position (X’s), for EDS patients (*dashed*) and healthy controls (*solid*). **c** Mean elliptical areas (cm^2^) collapsed across all hand-target locations. *Error bars* reflect standard error of the mean. **d** Mean axis length (cm) for the major, minor and summed axes. *Error bars* reflect standard error of the mean.

The bars in Figure 1a show that for both the EDS and control groups, subjects’ reach endpoints were quite accurate; reaches fell within 2 cm of the actual target-hand locations. Errors in proprioceptive localization for both groups are also depicted in Figure 1b as circles (mean endpoints) within the ellipses, relative to the “X” representing the location of the proprioceptive target. Horizontal errors were similar for both EDS (striped bars in Figure 1a; dashed circles in Figure 1b) and control subjects (solid bars and circles) [*F*(1, 20) = 2.50, *p* = 0.13], as were errors along the sagittal direction [*F*(1, 20) = 1.41, *p* = 0.25], and absolute displacement errors [*F*(1, 20) = 1.61, *p* = 0.22]. An interaction between group and (horizontal) target location revealed that leftward errors for left targets, and rightward errors for right targets, were larger for controls than EDS participants (whose reaches were shifted to the left for all targets) [*F*(1.15, 22.92) = 5.71, *p* = 0.022]. For sagittal errors, both groups tended to underestimate the distance of far proprioceptive targets, but not closer targets [*F*(1, 20) = 28.61, *p* < 0.001]. These results can be seen by comparing the circles in each ellipse to the “X”’s in Figure 1b.

### Precision

While EDS patients were as accurate as healthy controls, the larger dotted ellipses in Figure 1b suggest that they were not as precise at localizing their unseen left hand. EDS patients (dashed ellipses) showed greater scatter when localizing proprioceptive targets than healthy controls (solid ellipses). Specifically, the areas of the elliptic fits (Figure 1c) were significantly larger for EDS (striped bar; on average 8.33 cm^2^) than healthy controls (solid bar; on average 4.90 cm^2^) [*F*(1, 20) = 11.40, *p* < 0.001]. Likewise, the sum of the major and minor axes (shown in Figure 1d) was significantly larger for EDS patients than controls [*F*(1, 20) = 9.60, *p* < 0.001]. This increase in the sum of the axes reflected a significant increase in size of both axes (larger major [*F*(1, 20) = 5.28, *p* = 0.03], and minor axes [*F*(1, 20) = 18.82, *p* < 0.001]) for patients compared to healthy controls. This suggests that the increase in variance was not skewed in a particular direction. Although precision (both measured by elliptical area and sum-of-axes) was compromised at more peripheral target locations compared to the middle target location [*F*(1.17, 23.45) = 7.29, *p* = 0.001; *F*(1.24, 24.82) = 6.52, *p* = 0.013], this did not significantly vary with group [*F*(1.17, 23.45) = 0.69, *p* = 0.51; *F*(1.24, 24.82) = 0.44, *p* = 0.553].

### Chronic pain and proprioception

All EDS patients reported chronic pain, but at varying levels of severity. The mean PRI-R (pain rating index; ranked) for patients was found to be 31.33 and ranged from 3 to 61. Although some patients indicated they experienced more pain than others, we did not find a significant relationship between chronic pain scores and proprioceptive precision [*F*(1, 7) = 0.08, *p* = 0.79]. In other words, those with the largest elliptical areas did not have the highest PRI-R scores. This suggests that chronic pain is likely not contributing to the observed proprioceptive deficit shown in this study.

## Discussion

The goal of the present study was to explore proprioceptive sensitivity of the hand in EDS patients across space, and to see how their proprioceptive sensitivity compared to that of healthy controls. We used a robotic manipulandum to precisely place the non-dominant left hand at 6 different target-hand positions (near and far as well as left, centre and right) and measured subjects’ ability to localize the left hand using reaches with the seen right hand. Although EDS patients were as accurate as healthy controls, as shown by the similar horizontal and sagittal endpoint errors in Figure 1a (bars), and 1b (circles inside ellipses), patients were much less precise than healthy controls. Specifically, the magnitude of 2D scatter for proprioceptive localization errors, measured by 95% confidence ellipses (across 52 trials per target), for the patients were significantly larger (almost double) than those of the healthy controls. Overall, these results suggest EDS patients are impaired in their proprioceptive sensitivity.

For the most part, healthy individuals are quite good at localizing their unseen hand using proprioception (and efferent signals) (Lovelace 1989; Haggard et al. 2000; Jones et al. 2010, 2012a). While there are many ways to measure hand proprioception, one of the main methods involves subjects reaching to the current location of an unseen body part, in this case the hand (Lovelace 1989; Baud-Bovy and Viviani 1998; Haggard et al. 2000; Sarlegna and Sainburg 2007). Haggard et al. (2000) and Lovelace (1989) found that when subjects reached to the unseen location of their left index finger using a pen, they made localization errors of 1.74 and 1.77 cm, respectively. Results for proprioceptive-guided reaching from our lab (Jones et al. 2010, 2012a) also show that subjects are quite accurate in localizing their hand-target. When these hand-targets were radially displaced about 12 cm from the start position, from −30° to 120° (ranging horizontally by 20 cm), errors tended to systematically vary with the target angle. Nonetheless, average errors usually fell within 2 cm of the target site, for both the left and the right hands (Jones et al. 2010). When we tested a smaller number of proprioceptive target sites, at a similar distance, but located 5 cm left and right of the midline (including the midline), we found similar sized errors (Jones et al. 2012a). Again, the reach errors varied with the hand tested, (showed a hand-dependent bias) falling a couple centimetres on either side of the target site, consistent with the findings reported here. In the current study, and in Clayton et al. (2013), we find similar accuracy for both controls and EDS patients when localizing their unseen left hand. What differed between patients and controls was the precision of localizing the unseen hand: 95% error ellipses were about 4 cm^2^ for controls and double that for EDS patients.

While some studies have found that how well healthy people localize their unseen hand varies with the location in space (Jones et al. 2010; Wilson et al. 2010), in the current study, we found only small target-dependent effects for both accuracy and precision. For accuracy, the target-dependent pattern between groups was the same for sagittal errors or only mildly different for horizontal errors. For precision, both groups were slightly more variable when localizing their unseen hand at more peripheral locations compared to central localizations. However, as consistent with the overall effect, EDS patients showed almost twice the amount of scatter in both central and peripheral location as those of controls.

The larger variance that EDS patients show when localizing their felt hand, compared to controls, may reflect some impairments in their ability to reach. In our previous study (Clayton et al. 2013) we had EDS patients (half of them participated in both studies) reach to visual targets without visual feedback of their hand. Additional analyses from this earlier study revealed that EDS patients were just as accurate, but trended towards being less precise than controls when reaching to visual targets [*F*(1, 32) = 2.68, *p* = 0.11; *F*(1, 32) = 3.36. *p* = 0.08], by about 20%. Thus, the twofold increase in variance when reaching to the unseen hand in the current study compared to the 20% increase in variance when reaching (with the unseen hand) to a visual target in our previous study, suggests that the majority of the imprecision we find in this study reflects poorer hand proprioception. Nonetheless, it is possible that this impairment in hand proprioception could be what is driving the slightly larger variability in reaches to visual targets in the previous study where the reaching hand was not visible.

To reiterate, the current study shows that EDS patients were less precise (almost twice as variable) than controls at all target sites in the workspace. Specifically, they were just as variable at locating their unseen hand both when the hand was located along the body midline and when it fell 10 cm left and right of the midline (peripheral). This is somewhat different than our previous results where we found differences in precision (relative to controls) only at the peripheral locations. In that study, patients were twice as unsure of their hand’s position compared to controls, but only at more peripheral reference markers located 7.5 cm left and right of their body midline. The more global impairment in the current study may reflect a difference in the task; the current study involves subjects reaching to the proprioceptive target, while the previous study had them judge the felt location of the proprioceptive target relative to visual references. It could be that the way this sensory information is processed differs across task goals (e.g. Jones et al. 2012a)—we used a more perceptual task in Clayton et al. (2013), but a more goal-directed task in the current study. Alternatively, the difference across studies could be because this study involves localizing the non-dominant left hand, while Clayton et al. (2013) involved localizing the dominant right hand. People are usually poorer at perceiving the position of their non-dominant hand compared to their dominant (Haggard et al. 2000). Previous work in our lab (Jones et al. 2010) found that healthy controls were slightly less precise (about 20%) at reaching to the left-target hand compared to the right-target hand but found no difference in precision (the uncertainty range) between the two hands in a perceptual task similar to what was used in Clayton et al. (2013). Thus, if we used the right hand as a target in the current study, it is possible that overall, precision may have been slightly better for both controls and patients, but likely the patients would have still shown an impairment. In fact, we chose the non-dominant hand in this experiment in order to make the task slightly more challenging.

Another difference between our previous perceptual study and the current reach study was the relationship between joint hypermobility and proprioceptive impairment. In the previous study, we found a correlation between joint hypermobility, as measured by the Beighton scores, and the magnitude of the uncertainty ranges when perceptually judging the location of the dominant hand (in the peripheral locations). However, unlike our previous study, here the size of the deficit (variance in localization error) did not correlate with our measure of joint hypermobility (r^2^ = 0.21, *p* = 0.22). In other words, patients with lower Beighton scores were just as imprecise at proprioceptive localization as patients with higher Beighton scores. It can be argued that Beighton scores, which measure hypermobility at nine specific locales, are not the most ideal way to measure the magnitude of joint hypermobility (Fairbank et al. 1984). It is possible that we would have found different results had we measured hypermobility another way. Additionally, it could be that reaching to (rather than perceptually judging) the felt location of the non-dominant hand is challenging enough for even the least hypermobile patients to show deficits.

EDS patients are not the only special group that have shown impairments in proprioception. According to a review by Goble et al. (2009), several studies have shown that proprioceptive acuity decreases with age. These paradigms typically employ joint matching tasks where subjects are required to reproduce the perceived position of one joint with that of the other (Barrack et al. 1983; Adamo et al. 2007), or are asked to reproduce a joint angle from memory using the same or opposite arm (Kaplan et al. 1985; Adamo et al. 2007).

It is plausible that our observed deficit is being influenced by chronic pain, which is common in both EDS subtypes we studied (Sacheti et al. 1997; Voermans et al. 2010). Proprioceptive deficits have been observed in other chronic pain populations that do not exhibit joint hypermobility. For example, those with chronic low-back pain are found to have impaired lumbar proprioception (Gill and Callaghan 1998). However, pain does not need to be restricted to the body part being examined to reveal proprioceptive impairments. For example, those with neck pain are not able to reproduce elbow joint positions when their head is turned as well as healthy controls can (Knox et al. 2006). Thus, even though the patients in this study experienced pain in areas other than their left arm, it is possible that their pain could have influenced their proprioceptive judgements. However, this is not what we found; we found no correlation between chronic pain and proprioceptive precision. In other words, those with the worst proprioception (largest elliptical areas) were not those with the most chronic pain (highest PRI-R scores). It is possible that, had we examined more patients, such a relationship would reveal itself. Therefore we recommend gathering a much larger patient group to properly explore this relationship in the future.

It is unlikely that the proprioceptive deficits observed in EDS patients are due to sub-cortical impairments, like in the case of Parkinson’s disease (Lee et al. 2013). It is possible that these deficits are a result of probable abnormal collagen present in proprioceptors, but could also be due to repetitive stress-inducing injuries. It could be that these more downstream/peripheral components may be more related to the peripheral deterioration that is likely leading to proprioceptive impairments in the aged.

## Conclusion

In conclusion, although we found that EDS patients were just as accurate as controls when localizing their unseen, non-dominant left hand, they were not as precise. Specifically, patients showed twice as much scatter as controls when localizing this hand when it was placed at a variety of locations. However, we found no relationship between proprioceptive precision and chronic pain. These results suggest that EDS patients may experience different levels of proprioceptive sensitivity than healthy controls. Future work should explore how to improve proprioceptive sensitivity in the EDS population, which could reduce the frequency of accidental injuries in this group.

## Methods

### Subjects

Thirteen healthy age-matched control subjects (mean age 27 years, range 16–49, 5 females) and nine subjects with EDS (mean age 31 years, range 26–43, 8 females), all of whom were right handed, participated in the experiment described below. Control subjects were laboratory volunteers, or recruited from the Undergraduate Research Participant Pool at York University (and given course credit for their participation). Subjects in the patient group were recruited through EDS Canada’s GTA (Greater Toronto Area) Support Group. Patient clinical demographics are provided in Table 1. Four of the EDS patients were classic type (mean age 32 years, range 27–43, 3 females), while all of the others were hypermobility type (mean age 31, range 26–43, 5 females). All subjects had normal or corrected to normal vision. None of the EDS patients were on any medication known to affect their cognitive abilities during the experiment. Only patients with confirmed clinical diagnoses were admitted into the study. Joint hypermobility was measured using the Beighton criteria which rates patients’ hypermobility on a 9 point scale after performing 9 movements. Patients’ Beighton scores were obtained from genetic reports and, in all cases, were confirmed by the experimenter prior to testing. Chronic pain was measured using the McGill Pain Questionnaire, and a Pain Response Index (PRI-R) score was calculated for each subject by summing the rank value of each word chosen, as described in Melzack (1975). Patients read 20 sets of words, and were instructed to select the word that best described their pain (the least intense word is ranked 1) for each of the 20 dimensions, totalling a maximum of 78 points. If none of the words in a set applied, they made no selection (Melzack 1975).

**Table 1:** EDS clinical demographics

Subject	Age	Sex	Type	Beighton score	PRI-R
CM1	26	F	Hypermobility	6	11
CM2	27	F	Classic	7	61
CO	26	F	Hypermobility	8	57
RO	30	F	Hypermobility	6	3
MR	28	F	Hypermobility	7	23
BS	43	F	Classic	5	19
TS1	30	F	Classic	8	37
TS2	43	F	Hypermobility	8	43
TW	27	M	Classic	3	28

### General experimental setup

**Figure 2:**
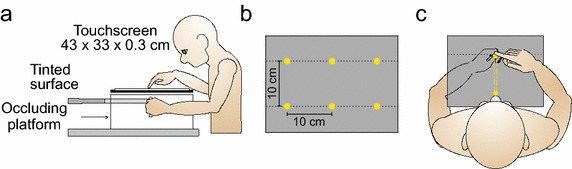
**a** Side view of the general experimental set-up. **b** Six locations served as start and final target sites for the non-dominant left hand. **c** The robotic manipulandum restricted active movement of the left target-hand along a straight path from one target site (start) to another target site (target position). Participants reached with their seen right hand to the felt location of the unseen left target-hand.

A view of the experimental setup is provided in Figure 2. Subjects sat on a height-adjustable chair in front of a 90-cm-high table. They were positioned so that they could comfortably reach to all areas of a transparent 43 cm (length) × 33 cm (width), 3-mm-thick horizontal touch screen panel (resolution of 4,096 × 4,096 pixels; Keytec, Garland, TX) placed on top of an occluding platform (Figure 2a). The touch screen was used to record all reach endpoints. A complete description of the methodology is reported elsewhere (Jones et al. 2012b).

Six sites served as the proprioceptive target locations (Figure 2b). These sites were spaced 10 cm apart and arranged in two lines (3 sites/line and 10 cm between the two lines). The closest three target sites were 23 cm from participants (bottom line of targets in Figure 2b). Subjects were instructed to grasp the vertical handle of a modified two-joint robotic manipulandum (Interactive Motion Technologies, Cambridge, MA, USA) with their left target-hand in such a way that their thumb rested on top of the robot handle (1.4 cm in diameter); the handle was just above waist level (Figure 2a). On each trial, the robotic manipulandum was programmed to restrict subjects’ active movement of their left hand along a straight path from one of the six target-hand locations to one of the five remaining target-hand locations (dashed lines in Figure 2c; see Cressman and Henriques (2009) for details about active placement of the target-hand). On each trial, participants were asked to reach to the felt location of their left thumb located at one of the six sites (Figure 2c). For convenience, the term target-hand will be used in place of target-thumb.

The manipulandum was occluded by a tinted translucent Plexiglas platform (on which the transparent touch screen panel was fixed), which was located 2 cm above the height of the target-hand (Figure 2a). Once the room lights were turned off, subjects were not able to see their left target-hand or forearm. A cloak was used to cover the subject’s left upper arm and shoulder to ensure that no additional visual information concerning hand or arm position could be used at any point throughout the testing sessions (cloak not shown in Figure 2a). Subjects could see their right reaching-hand.

Subjects reached with their right (dominant) hand to their unseen left target-hand (Figure 2c). Each session began with the left target-hand at the bottom-center target-hand location (Figure 2b). Subjects first reached with their right hand/index finger to the felt location of the left target-hand in this initial location. A tone indicated to subjects’ that they made contact with the touch screen. Subjects then returned their right reaching-hand to the right of their body and actively pushed the robotic manipulandum using their left target-hand (guided along the robot-constrained pathway, illustrated as a yellow rectangle in Figure 2c) from this starting location to one of the five remaining target sites. Once the left target-hand arrived at its final location, a tone prompted subjects to once again reach to the left target-hand at this new target site, making contact with the touch screen with the index finger of their right hand (Figure 2c). Subjects then returned their right reaching-hand to the right of their body, and the left target-hand was actively guided to the next final target-hand location. Therefore, the final position of the left-target hand for each reach trial served as the starting position of the left target-hand for the subsequent trial. To limit proprioceptive drift, the left target-hand began in the bottom-center start location twice as many times as in any other starting position. On 50% of these trials, the left target-hand was illuminated using three white light emitting diodes (LEDs), and was therefore visible, for 1 s (Wann and Ibrahim 1992; Desmurget et al. 2000; Brown et al. 2003). The illumination of the target-hand in this bottom-center location occurred prior to reach onset of the right hand. The left target-hand was not illuminated in any other location in this task. Trials in which the left target-hand was visible in the bottom-center location were not included in the analysis.

Each subject made 52 reaches to the left target-hand for each of the 35 start and final target position combinations (including those combinations when the target-hand was illuminated in the bottom-center start position) for a grand total of 1,820 trials. Two sessions were used to collect 6 blocks of data across 2 days of testing. Each participant also completed a baseline reaching task at the end of each experimental block. The baseline task consisted of five reaches to the continuously visible left target-hand for each start and final target position combination. Horizontal and sagittal reach errors were calculated by taking the reach endpoint, as recorded by the touch screen, for each reaching trial and subtracting this baseline average reach endpoint for each start and target position pairing. Precision (or variability) of the proprioceptive-guided reaches was examined by fitting 95% error ellipses around reach endpoints for each final target position, for each subject. The area of the ellipses, as well as the sum of the major and minor axes, was used to compare precision of locating the unseen left hand across groups and proprioceptive target positions. While area of the ellipses provides a common and intuitive assessment of variance, using the length of the axes provides a robust measure which is less sensitive to outliers.

### Data analysis

To assess proprioceptive accuracy in EDS patients and healthy controls, we compared horizontal and sagittal errors using a mixed ANOVA that included group (EDS vs. healthy) as a between-groups factor and proprioceptive target location (target-hand positions that were near-left, near-centre, near-right, far-left, far-centre and far-right) as a within-groups factor. To assess differences in precision of hand localization between the two groups, we ran similar 2 × 6 mixed ANOVAs on elliptical error and the individual and sum of elliptical axes. All ANOVA results are reported with Greenhouse-Geisser corrected *p* values to compensate for violations of sphericity. Differences with a probability of *p* ≤ 0.05 were considered significant. Pairwise comparisons with Bonferroni correction were used to determine the locus of these differences. Finally, we ran regression analyses to explore the relationship between chronic pain and proprioceptive precision, as well as that of joint hypermobility and proprioceptive precision.

## Abbreviations

EDS: Ehlers–Danlos syndrome
HMS: hypermobility syndrome
GTA: greater Toronto area
PRI-R: Pain Response Index, ranked
LEDs: light emitting diodes
